# Improved adherence to test, treat, and track (T3) malaria strategy among Over-the-Counter Medicine Sellers (OTCMS) through interventions implemented in selected rural communities of Fanteakwa North district, Ghana

**DOI:** 10.1186/s12936-022-04338-9

**Published:** 2022-11-05

**Authors:** Olajoju Temidayo Soniran, Benedicta Ayiedu Mensah, Ndong Ignatius Cheng, Benjamin Abuaku, Collins Stephen Ahorlu

**Affiliations:** 1 Department of Epidemiology, Noguchi Memorial Institute for Medical Research, University of Ghana, Accra, Ghana; 2 Department of Science Laboratory Technology, Akanu Ibiam Federal Polytechnic, Unwana, Afikpo, Ebonyi State Nigeria; 3 Department of Biochemistry, Faculty of Science, Catholic University of Cameroon, Bamenda, Cameroon

**Keywords:** Malaria, Private medicine retailers, Over-the-counter medicine sellers, Test, treat, and track (T3), Implementation research

## Abstract

**Background:**

Prompt diagnosis and treatment of malaria prevents a mild case from developing into severe disease and death. Unfortunately, parasitological testing of febrile children is greater in the public and formal private sector than in the informal private sector where many patients with malaria-like symptoms first seek treatment. This study was aimed at improving implementation of the T3 policy among OTCMS using some interventions that could be scaled-up easily at the national level.

**Methods:**

Interventions were evaluated using a two-arm, cluster randomized trial across 8 rural communities (4 clusters per arm), in two adjacent districts of Ghana. A total of 7 OTCMS in the intervention arm and 5 OTCMS in the control arm in the selected communities participated in the study. Five interventions were implemented in the intervention arm only. These were acquisition of subsidized malaria rapid diagnostic test (RDT) kits, training of OTCMS, supportive visits to OTCMS, community sensitization on malaria, and introduction of malaria surveillance tool. The primary outcome was the proportion of children under 10 years with fever or suspected to have malaria visiting OTCMS and getting tested (using RDT) before treatment. Secondary outcomes included OTCMS adherence to national malaria treatment guidelines and the recommended RDT retail price. Outcomes were measured using mystery client (an adult who pretends to be a real patient) surveys supplemented by a household survey. Proportions were compared using chi-square test or Fisher exact test.

**Results:**

Following deployment of interventions, mystery client survey showed that OTCMS’ adherence to malaria protocol in the intervention arm increased significantly (p < 0.05) compared to the control arm. Household surveys in the intervention arm showed that caregivers self-treating their children or visiting drug vendors significantly decreased in favour of visits to OTCMS shops for treatment (p < 0.001). End-line malaria testing rate was higher compared with the baseline rate, though not statistically significant (30.8% vs 10.5%; p = 0.1238). OTCMS in the intervention arm also adhered to the subsidized RDT retail price of GHc2.40.

**Conclusion:**

Interventions targeting OTCMS in rural communities have the potential of improving adherence to the T3 malaria policy and subsequently improving management of uncomplicated malaria in Ghana.

*Trial registration*: ISRCTN registry ISRCTN77836926. Registered on 4 November 2019.

## Background

Malaria continues to account for high mortality and morbidity globally especially in sub-Saharan African countries. In 2020, global malaria deaths was estimated at 627,000 and World Health Organization (WHO) African Region accounted for 96% of these deaths. Children under 5 years and pregnant women are the most affected [1]. Most malaria related deaths can be averted if cases are detected on time, diagnosed promptly, and treated according to recommended malaria management guidelines [2].

The WHO recommends a confirmatory blood test for all suspected malaria cases and a prescription of artemisinin-based combination therapy (ACT) for those who test positive [3]. The deployment of malaria rapid diagnostic tests (RDTs) is a useful measure in the management of uncomplicated malaria particularly in highly endemic rural settings, where microscopy is a challenge [4]. In the public health care sector, procurement of RDTs has increased significantly across sub-Saharan Africa [5, 6]. Sadly, the availability and use of RDTs is low in the private medicine retail (PMR) sector where most patients with malaria-like symptoms seek treatment [7].

In Ghana, Over-the-Counter Medicine Sellers (OTCMS) a subsidiary of the PMR, are usually the first point of call for patients because there are no consultation fees, little or no waiting times, and patients’ preference for self-medication. OTCMS are regulated by the Pharmacy Council of Ghana and limited by the Pharmacy Act of Ghana to selling only class C (over the counter) medicines which includes antimalarial drugs. As of 2012, out of over 10,000 OTCMS in Ghana, 233 had been accredited by the National Health Insurance Authority [8, 9]. Although, previous studies in Ghana and across sub-Saharan Africa had reported varying uptake of RDT among OTCMS, adherence to RDT-negative test result is very low [10] and community members still held the view that RDT-negative results did not mean ‘no malaria illness’ and would therefore use ACT [11, 12].

Given the importance of OTCMS as a first source of care and antimalarial treatment in Ghana, scaling up RDTs in these outlets will achieve universal access to prompt parasite-based diagnosis prior to treatment. However, evidence to guide decisions on how and where to scale up RDTs amongst OTCMS is currently lacking [7]. Hence, objective of this study was to evaluate the combined effectiveness of provider and community interventions on RDT testing rates in the study areas.

## Methods

### Study area

The study was conducted in Fanteakwa North and Fanteakwa South districts in the eastern region of Ghana. The two districts were previously one (Fanteakwa district) until March 2018 when the southern part of the district was split off to create Fanteakwa South district and the remaining part renamed Fanteakwa North district. The area has been described elsewhere [10]. Briefly, Fanteakwa North district has 1 hospital, no health centre, 1 clinic, 31 community health-based planning services (CHPS) compounds, and 28 OTCMS, while the Fanteakwa South has no hospital, 2 health centres, 1 clinic, 15 CHPS compounds, and 19 OTCMS. Begoro, the capital town of Fanteakwa North district acted as a buffer between the two districts in this study.

### Study design

This implementation research study was conducted between September 2019 and November 2020. A quantitative approach using household questionnaire surveys targeting caregivers of children under 10 years in the intervention arm only and mystery clients visiting the OTCMS in both intervention and control arms. Interventions were evaluated using a two-arm (intervention and control), cluster randomized trial across 8 rural clusters (4 clusters per arm), in two adjacent districts of Ghana. This study evaluated the combined effectiveness of different interventions. The intervention arm has 8 clusters, and out of these, 4 were randomly selected using a computer-generated list. The control arm had 4 clusters, and all these were included in the study. An urban sub-district (Begoro) in the intervention district acted as a buffer between the two arms. A total of 7 OTCMS in the intervention arm and 5 OTCMS in the control arm participated in the study. The aim of the study was to evaluate the combined effectiveness of provider and community interventions on the proportion of children under 10 years who receive treatment for malaria without testing at OTCMS as well as the level of service provider (OTCMS) adherence to malaria case management guidelines. This was accomplished by comparing malaria RDT testing rates between pre-intervention and post-intervention periods.

### Study procedures

The study had 4 phases. These are preparatory, baseline, intervention, and evaluation.

#### Preparatory phase

In the preparatory phase, meetings were scheduled with relevant stakeholders including the district and regional health directorates, the National Malaria Control Programme (NMCP), relevant non-governmental organizations such as Strengthening Health Outcomes through the Private Sector (SHOPS), OTCMS, and traditional leaders in the selected communities. Houses in the selected clusters were mapped and lists of households with children under 10 years old were generated with corresponding GPS coordinates.

#### Baseline phase

The baseline phase involved conducting community entry and household surveys in each of the intervention clusters, and in-depth interviews of OTCMS in both the intervention and control clusters.

*Baseline household survey*: The survey was conducted in the intervention arm to document preintervention malaria testing rates among children under 10 years visiting OTCMS for malaria treatment in the past 1 month preceding the survey.

*In-depth interviews*: In-depth interviews with OTCMS in the selected clusters/communities in both study arms were conducted to determine possible factors preventing the effective management of malaria at their level.

The findings of the baseline phase had been reported elsewhere [13].

#### Intervention phase

The interventions implemented in this study included:

- Provision of subsidized RDT kits for OTCMS- The RDT kits were obtained from the National Malaria Control Programme and supplied to OTCMS at no cost. The OTCMS were instructed to test their febrile clients at a subsidized rate of GH¢2.40/kit (~ $0.44), a means of providing incentive for the OTCMS.
- Training of OTCMS: A 2 day training workshop was conducted for OTCMS in the selected intervention clusters on malaria management protocol, appropriate treatment, and follow-ups on their clients. Malaria management protocol in this study is referred to when:
- OTCMS sight every patient suspected of malaria for examination and diagnosis. This includes requesting caregivers of febrile children (visiting the OTCMS for prescription without the child physically present) to bring the child to his/her outlet.
- OTCMS conducts a malaria blood test on patients suspected of uncomplicated malaria before prescription of medicine.

(iii) Quarterly supportive visits to OTCMS: the OTCMS in the intervention arm were visited quarterly during the implementation phase to monitor and assess their malaria management practices. The skills acquired during the earlier training workshop was reinforced, and technical guidance provided on challenges experienced.
(iv) Community sensitization on malaria focusing on the T3 strategy: the intervention communities were sensitized on malaria and importance of demanding malaria testing before treatment. Community health workers and town criers (‘*Gongong’*) were engaged to carry out this activity at churches, mosques, community durbars and on market days.
(v) Introduction of malaria surveillance tool for use by OTCMS: the OTCMS were enlightened and trained on how to keep accurate record of all suspected malaria cases attended to using this tool. The communities were also sensitized on the surveillance tool.

#### Evaluation phase

The primary outcome was measured using mystery client surveys and end-line household survey conducted in the evaluation phase.

*Mystery client survey*: Mystery client surveys were used to evaluate OTCMS conduct in implementing the T3 strategy. Mystery client data collection covered 9th to 11th months of the intervention period. The mystery clients also assessed the process of RDT use in the two study arms. A total of 13 mystery clients were recruited and given intensive training (including practical sessions) over 3 days on the clinical scenario, how to conduct and interpret a malaria blood test using RDT, and how to fill the assessment checklist/form. Each OTCMS was visited twice a month by a different mystery client for 3 months (August 2020–October 2020). Two different clinical scenarios were presented by the mystery clients during the visits to the OTCMS:

(i) Pretending to have fever in the past 24 h.
(ii) A caregiver seeking medical care on behalf of his/her febrile child (who is not physically present at the OTCMS shop at the time of the visit).

The mystery client then observed the OTCMS’ response whether RDT will be proposed or not before treatment. The mystery client filled the assessment checklist based on outcome of his/her visit, but when out of sight of the OTCMS.

A total of 72 visits (42 and 30 visits in the intervention and control arms respectively) were conducted in the mystery client survey.

*End-line household survey*: The survey was conducted in the intervention arm to document post-intervention malaria testing rates among children under 10 years visiting OTCMS for malaria treatment in the past 1 month preceding the survey. A semi-structured questionnaire was developed, pre-tested for validity and administered by trained data collectors to respondents (caregivers/mothers of children under 10 years old). The questionnaire covered topics such as: Socio-demographics of respondent; knowledge of malaria and its transmission; history of fever among children under 10 years in the past 1 month; caregiver’s treatment-seeking behaviour; and insecticide-treated bed net usage.

### Data collection

*Data collection team* The study employed data collectors (school teachers) from each community who could conduct the mystery client survey. The data collectors were trained on the purpose of the study and questionnaire administration. Following the training, the data collection tools were pretested, and adjustments were made where necessary.

### Data analysis

Data were double entered and cleaned using Microsoft Access 2010 (Microsoft Inc., Redmond, Washington) and analysed using STATA version 11.0. Variables of interest were summarized using descriptive statistics. Proportions between groups were compared using chi-square test or fisher exact test (p ≤ 0.05 considered statistically significant).

## Results

### Socio-demographic characteristics of study OTCMS, caregivers and children under 10 years old

A total of 12 OTCMS service providers participated in the study. These included 7 OTCMS and 5 OTCMS in the intervention and control arms, respectively. Majority were males (75%); aged 20–30 years (50%); had about 10 years of experience (50%); married (58.7%); with Senior High School educational background (75%); Christians (91.7%); and practiced farming as an additional occupation (58.3) (Table 1).

**Table 1:** Demographic characteristics of OTCMS service providers that participated in the study

Characteristics	Both arms n (%)	Intervention arm n (%)	Control arm n (%)
**Total OTCMS**	12 (100)	7 (100)	5 (100)
**Gender**			
Male	9 (75)	6 (85.7)	3 (60)
Female	3 (25)	1 (14.3)	2 (40)
**Age** **(years)**			
20–30	6 (50.0)	2 (28.6)	4 (80)
31–40	1 (8.3)	1 (14.3)	0 (0)
41–50	1 (8.3)	1 (14.3)	0 (0)
51–60	2 (16.7)	1 (14.3)	1 (20)
> 60	2 (16.7)	2 (28.6)	0 (0)
**Years of experience**			
1–10	6 (50.0)	2 (28.6)	4 (80)
11–20	5 (41.7)	4 (57.1)	1 (20)
21–30	1 (8.3)	1 (14.3)	0 (0)
**Marital status**			
Single	5 (41.7)	2 (28.6)	3 (60)
Married	7 (58.3)	5 (71.4)	2 (40)
**Level of education**			
Junior high school	1 (8.3)	0 (0)	1 (20)
Senior high school	9 (75.0)	5 (71.4)	4 (80)
Tertiary	2 (16.7)	2 (28.6)	0 (0)
**Religion**			
Christianity	11 (91.7)	6 (85.7)	5 (100)
Islam	1 (8.3)	1 (14.3)	0 (0)
**Additional occupation**			
None	4 (33.3)	2 (28.6)	2 (40)
Farming/fishing	7 (58.3)	4 (57.1)	3 (60)
Petty trader	1 (8.3)	1 (14.3)	0 (0)

Caregivers of children under 10 years that participated in the household survey were 291 and 346 in the intervention and control arms, respectively. Majority were females (94.7%); aged 18–30 years; married (79.1%); with primary educational background; Christians (94.5%); and practiced either petty trading or farming (35.3% and 34.2%, respectively). There was a significant difference (p < 0.05) in the proportions of socio-demographic variables considered in the study between the two arms (Table 2).

**Table 2:** Demographic characteristics of caregivers of children under 10 years in the end-line household survey

Characteristics	n (%)	Intervention Arm	Control Arm	X2, p value
Total caregivers	637 (100)	291 (100)	346 (100)	
**Sex** n (%)				29.95, < 0.00001
Males	34 (5.3)	31 (10.7)	3 (0.9)	
Females	603 (94.7)	260 (89.3)	343 (99.1)	
**Age** (years) n (%)				15.09, 0.0045
< 18	3 (0.5)	2 (0.7)	1 (0.3)	
18–30	264 (41.4)	105 (36.1)	159 (46.0)	
31–40	214 (33.6)	97 (33.3)	117 (33.8)	
41–50	106 (16.6)	53 (18.2)	53 (15.3)	
> 50	50 (7.8)	34 (11.7)	16 (4.6)	
**Marital status n (%)**				10.07, 0.039
Single	79 (12.4)	29 (10.0)	50 (14.5)	
Married	504 (79.1)	238 (81.8)	266 (76.8)	
Separated	18 (2.8)	5 (1.7)	13 (3.8)	
Divorced	17 (2.7)	6 (2.1)	11 (3.2)	
Widowed	19 (3.0)	13 (4.5)	6 (1.7)	
**Educational level, n (%)**				54.85. < 0.00001
None	111 (17.4)	79 (27.2)	32 (9.2)	
Primary	148 (23.2)	81 (27.8)	67 (19.4)	
Junior High	317 (49.8)	114 (39.2)	203 (58.7)	
Senior High	43 (6.7)	10 (3.4)	33 (9.5)	
Vocational training	1 (0.2)	0 (0.0)	1 (0.3)	
University	17 (2.7)	7 (2.4)	10 (2.9)	
**Religion, n (%)**				9.9463, 0.0069
None	8 (1.3)	6 (2.1)	2 (0.6)	
Christianity	602 (94.5)	266 (91.4)	336 (97.1)	
Islam	27 (4.2)	19 (6.5)	8 (2.3)	
**Primary occupation, n (%)**				114.70, < 0.00001
Unemployed	66 (10.4)	36 (12.4)	30 (8.7)	
Farming/ Fishing	218 (34.2)	158 (54.3)	60 (17.3)	
Petty trading	225 (35.3)	55 (18.9)	170 (49.1)	
Civil servant/ Government official	23 (3.6)	10 (3.4)	13 (3.8)	
Artisan	100 (15.7)	31 (10.7)	69 (19.9)	
Other	5 (0.7)	1 (0.3)	4 (1.2)	

A total of 1,225 children under 10 years were under the care of the caregivers. These included 574 and 651 children in the intervention and control arms respectively. Majority were males (53.2); aged 6–10 years (54.3%); and biological son/daughters of their caregivers (91.1%) (Table 3).

**Table 3:** Demographic characteristics of children  ≤ 10 years under the caregivers in the end-line household survey

Characteristics	Both arms n (%)	Intervention Arm n (%)	Control Arm n (%)	X2, p value
Total children	1,225 (100)	574 (100)	651 (100)	
**Gender** n (%)				
Males	652 (53.2)	312 (54.4)	340 (52.2)	0.47, 0.491
Females	573 (46.8)	262 (45.6)	311 (47.8)	
**Age** (years) n (%)				0.03, 0.872
< 5	560 (45.7)	261 (45.5)	299 (45.9)	
6–10	665 (54.3)	313 (54.5)	352 (54.1)	
**Age (mean + SD)**		5.03 ± 2.83	4.79 ± 2.72	t = −1.4810, 0.9306
**Caregiver-child relationship, n (%)**				16.14, 0.001
Son/daughter	1116 (91.1)	508 (88.6)	608 (93.5)	
Grandchild	91 (7.4)	60 (10.5)	31 (4.8)	
Niece/nephew	16 (1.3)	6 (0.9)	10 (1.5)	
Not related	1 (0.1)	0 (0.0)	1 (0.2)	

### Caregivers’ treatment-seeking behaviour

**Fig. 1:**
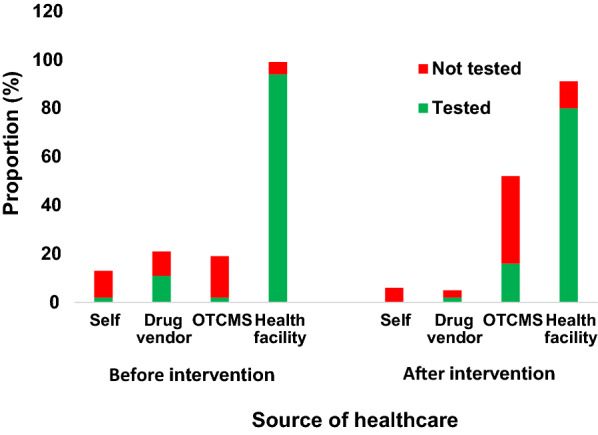
Treatment-seeking behaviour of caregivers and proportion of febrile children tested before treatment

In the intervention arm only, the proportion of caregivers self-treating their febrile children or visiting drug vendors (drug peddlers) during the pre-intervention period (baseline) significantly decreased in favour of visits to OTCMS shops after the deployment of interventions (endline) (p < 0.001) (Fig. 1).

### Caregivers’ report on management of fever in children

In the intervention arm only, the proportion of children reported to have had fever within 30 days prior to the survey was 26.8% (154/ 574). Many of these febrile children (59.1%) were reported to have been sent to a government health facility whilst 33.8% (52/154) were sent to OTCMS for care. A total of 80 of the children sent to a government health facility (87.9%; 95% CI 88.1–98.1) were reported to have received a malaria test before treatment while 16 of the children sent to OTCMS (30.8%; 95% CI 1.8–34.5) were reported to have received a malaria test before treatment (Table 4). Children who received an ACT without a malaria test were 10(11.6%) at the government health facility and 24(60%) at the OTCMS. Post-intervention, malaria testing rate by OTCMS was higher compared to pre-intervention period, though not statistically significant (30.8% vs 10.5%; p = 0.1238). Prescription of ACT to children not diagnosed also reduced after the intervention but not statistically significant (60% vs 80%; p = 0.2385) (Table 5).

**Table 4:** Caregivers’ report on diagnosis and treatment of febrile children  < 10 years old (intervention arm only)

Treatment	N (%)	Sources of treatment and diagnosis							
		Self n (%)		Drug vendor n (%)		OTCMS n (%)		Health centre n (%)	
		Tested	Not tested	Tested	Not tested	Tested	Not tested	Tested	Not tested
Herbs	4 (2.6)	0 (0.0)	3 (100)	0 (0.0)	0 (0.0)	0 (0.0)	1 (100)	0 (0.0)	0 (0.0)
ACT	132 (85.7)	0 (0.0)	1 (100)	2 (40)	3 (60)	16 (40)	24 (60)	76 (88.4)	10 (11.6)
Non-antimalarials	18 (7.1)	0 (0.0)	2 (100)	0 (0.0)	0 (0.0)	0 (0.0)	11 (100)	4 (80)	1 (20)
		0 (0.0)	6 (100)	2 (40)	3 (60)	16 (30.8)	36 (69.2)	80 (87.9)	11 (12.1)
Grand Total	154 (100)	6 (3.9)		5 (3.2)		52 (33.8)		91 (59.1)	

*ACT* Artemisinin-based Combination Therapy; () malaria testing/non-testing rate

**Table 5:** Caregivers’ reports on malaria testing rate and treatment of their febrile children by OTCMS

	Before intervention n (%)			After intervention n (%)		
	Tested	Not tested	Total	Tested	Not tested	Total
Herbs	0 (0.0)	0 (0.0)	0 (0.0)	0 (0.0)	1 (2.7)	1 (100)
ACT	2 (20)	8 (80)	10 (100)	16 (40)	24 (60)	40 (100)
Non-antimalarials	0 (0.0)	9 (100)	9 (100)	0 (0.0)	11 (30.6)	11 (100)
Total	2 (10.5)	17 (89.5)	19 (100)	16 (30.8)	36 (69.2)	52 (100)

*ACT* Artemisinin-based Combination Therapy

### OTCMS’ adherence to malaria protocol

Mystery client surveys in the two study arms showed that the total adherence to malaria protocol by OTCMS was 55.6%. However, adherence to malaria protocol by OTCMS (66.7%) in the intervention arm was significantly (p < 0.05) higher compared to the control arm (40%) (Table 6).

**Table 6:** OTCMS’ adherence to malaria protocol during mystery client visits

Adherence to protocol	Number of visits			X^2^, p value
	Intervention arm	Control arm	Total	
Yes	28 (66.7)	12 (40)	40 (55.6)	5.04, 0.0247
No	14 (33.3)	18 (60)	32 (44.4)	
Total	42 (100)	30 (100)	72 (100)	

### Malaria testing rate and prescription of medicine by OTCMS’

Mystery client survey also showed that malaria testing rate in the intervention arm (38.1%) was higher than in the control arm (23.3%) though not statistically significant (p = 0.1853). The proportion of anti-malarial drug (ACT) prescribed to clients ‘not tested’ in the intervention arm was 64.3% compared to the control arm (75%) though not statistically significant (p > 0.05) (Table 7).

**Table 7:** Malaria testing rate and prescription of medicine to mystery clients by OTCMS

	Intervention				Control			
	Tested		Not tested	Total	Tested		Not tested	Total
	Positive	Negative			Positive	Negative		
ACT	3 (21.4)	2 (14.3)	9 (64.3)	14 (100)	3 (18.8)	1 (6.3)	12 (75)	16 (100)
Quinine	0 (0)	0 (0)	1 (100)	1 (100)	0 (0)	0 (0)	2 (100)	2 (100)
Non-antimalarial	1 (7.7)	8 (61.5)	4 (30.8)	13 (100)	0 (0)	3 (42.9)	4 (57.1)	7 (100)
No drug prescribed	0 (0)	2 (14.3)	12 (85.7)	14 (100)	0 (0)	0 (0)	5 (100)	5 (100)
Grand total	16 (38.1)		26 (61.9)	42 (100)	7 (23.3)		23 (76.7)	30 (100)

### Assessment of OTCMS’ conduct of RDT and interpretation of results

Majority of OTCMS in the intervention arm followed the recommended steps while conducting the malaria test for their clients as 75% wore a glove before conducting the malaria test; labelled client’s RDT cassette correctly (75%); used the recommended finger (68.8%); disposed of the lancet correctly (68.8%); wiped the first blood using cotton wool (87.5%); allowed an average of 17.1 min to lapse before reading the test result; and interpreted the results correctly (100%) (Table 8).

**Table 8:** Assessment of OTCMS’ conduct of RDT and interpretation of results

Parameters	Intervention arm n (%)	Control arm n (%)
Conducted a malaria blood test	16 (100)	7 (100)
Wore hand gloves before conducting test	12 (75)	0 (0)
Indicated the right labelling on cassette	12 (75)	3 (42.9)
Used recommended finger	11 (68.8)	2 (28.6)
Disinfected finger	16 (100)	7 (100)
Lancet disposed correctly	11 (68.8)	5 (71.4)
First blood wiped using cotton wool	14 (87.5)	3 (42.9)
Used the right amount of blood	11 (68.8)	6 (85.7)
Blood placed in the appropriate well of RDT kit	16 (100)	7 (100)
Blood collecting device disposed correctly	10 (62.5)	4 (57.1)
Buffer placed in appropriate well	16 (100)	7 (100)
Recommended amount of buffer	15 (93.8)	7 (100)
Checked time immediately after adding buffer	5 (31.3)	0 (0)
Disposed other waste materials correctly	12 (75)	6 (85.7)
Average time elapsed before reading test results (minutes)	17.1	9.4
Interpreted the results correctly	16 (100)	5 (71.4)

## Discussion

The OTCMS are often the first source of care for healthcare seekers in the communities and hence, have the potential for increased universal access to prompt parasite-based diagnosis prior to treatment, if RDTs are introduced and scaled-up in this sector. However, evidence to guide decisions on introduction and scaling-up of RDTs among OTCMS is lacking [14]. This study presents findings indicating the possibility of scaling-up RDTs among the OTCMS.

Health-seeking behaviour for malaria treatment is important in the success of preventing malaria related mortality [15, 16]. Before implementation of interventions, caregivers of febrile children practiced self-treatment and patronized drug peddlers [13]. But this attitude changed because of interventions implemented which included sensitization on malaria and increased malaria testing using RDT before prescription of medicine by OTCMS.

The interventions implemented also improved OTCMS’ adherence to malaria protocol. The existing practice of OTCMS on management of malaria was observed in the control arm. Prescription of antimalarials by OTCMS to caregivers of febrile children without the child physically present for a malaria blood test to be conducted on him or her was a prevalent practice. For febrile adults visiting the OTCMS, the recommended malaria test before prescription of medicine was optional based on patient’s ability to afford the cost of testing and sometimes availability of RDT kit in stock. However, with the introduction and regular supply of RDT kits as part of the interventions implemented in the present study, malaria testing rate increased, and most clients could afford at least 1 Ghanaian cedi ($0.17) for a malaria test (unpublished report). The malaria testing rate reported in the present study is between 30.8% and 38.1%, but in a similar study (except for monthly supervision of healthcare providers and no promotional activities) conducted in Myanmar, RDT uptake was 59% [17]. Generally, previous studies with more intensive interventions produced better outcomes, but it is unclear whether such efforts could be maintained or scaled up to national level [7].

During mystery client survey, the increased malaria testing rate reported though not statistically significant compared to the control arm may be due to the outbreak of coronavirus disease 2019 (COVID-19) pandemic at the middle of the study period. In two communities, anecdotal reports showed that client patronage of OTCMS reduced during this period because of misconception that malaria blood test is being used to conduct COVID-19 surveillance. However, it is noteworthy that the quantity of anti-malarials prescribed by OTCMS in the intervention arm reduced (though not significantly) compared to the control arm. This shows that if malaria RDT is scaled up among OTCMS along with proper training and monitoring, a lot of anti-malarials prescribed to patients not having malaria in previous practice will be saved for confirmed malaria positive patients.

It is also noteworthy that the OTCMS in the intervention arm applied the skills acquired in their training while conducting RDT compared to their counterparts. They were conscious of protecting themselves and their clients against health risks, hence hand gloves were worn before conducting the malaria test. A higher proportion of the OTCMS also labelled individual patient’s cassettes to avoid confusion and misdiagnosis especially when attending to more than one patient at a time. The recommended finger (closest to the index finger) was pricked for collection of blood samples as recommended by the health authorities. Majority of the trained OTCMS also wiped off the first blood after finger prick before collecting the blood sample for the test, placed the right amount of blood and buffer in the appropriate places of the device and waited for an average time of 17 min though the manufacturer’s recommended time was 20 min. Majority of them also disposed the lancets inside improvised empty beverage cans which were buried under the ground or burnt with fire, along with other waste materials (like used cotton wool and used kits). They acted this way because of the knowledge that such materials constitute hazards to the environment. Interpretation of RDT results and adherence was also encouraging as majority of the patients that tested negative were not given any anti-malarials but directed to the nearest health facility. Observation also showed that OTCMS who did not engage in other businesses outside their profession at their outlet/shop location conducted better malaria blood tests. The ability of the OTCMS to adhere to these guidelines improved the quality of the RDT conducted compared to their counterpart in the control arm.

## Conclusions

Currently, the proposed scale up of RDTs among OTCMS is limited by controversies and lack of evidence to guide decisions on how and where to scale up RDTs [7]. Some of the concerns of public officials include fear that OTCMS may not treat patients according to malaria guidelines, may not adhere to test results, and handle hazardous wastes improperly which may lead to the spread of infectious illnesses [18–20]. It is noteworthy that the present study provided evidence on some of these gaps which might help policy makers in making informed decision.

With proper supervision and monitoring, scalable interventions targeting OTCMS in rural communities have the potential of improving adherence to the T3 malaria policy and subsequently improving management of uncomplicated malaria in Ghana.

## Data Availability

The datasets supporting the conclusions of this article are available in the Department of Epidemiology, Noguchi Memorial Institute for Medical Research and are available through the corresponding author on reasonable request.

## Abbreviations

OTCMS: Over-the-counter medicine sellers
T3: Test, treat, and track
RDT: Rapid diagnostic test
WHO: Word Health Organization
ACT: Artemisinin-based combination therapy
PMR: Private medicine retailers
CHPS: Community health-based planning services
NMCP: National malaria control programme
SHOPS: Strengthening health outcomes through the private sector
COVID-19: Coronavirus disease 2019
